# Trends and Seasonality of Information Searches Carried Out through Google on Nutrition and Healthy Diet in Relation to Occupational Health: Infodemiological Study

**DOI:** 10.3390/nu13124300

**Published:** 2021-11-28

**Authors:** Ruben Palomo-Llinares, Julia Sánchez-Tormo, Carmina Wanden-Berghe, Javier Sanz-Valero

**Affiliations:** 1 Department of Public Health and History of Science, School of Medicine, Miguel Hernandez University, 03550 Alicante, Spain; palomo.rub@gmail.com; 2 International Virtual Center for Nutrition Research (CIVIN), 03540 Alicante, Spain; jsancheztorm@gmail.com; 3 Foundation for the Promotion of Health and Biomedical Research in the Valencian Region (FISABIO), Health and Biomedical Research Institute of Alicante (ISABIAL), 03010 Alicante, Spain; carminaw@telefonica.net; 4 Carlos III Health Institute, National School of Occupational Medicine, 28029 Madrid, Spain

**Keywords:** nutrition, healthy diet, occupational health, access to information, Google trends, relative search volume

## Abstract

This study aimed to analyze and relate the population interest through information search trends on Nutrition and Healthy Diet (HD) with the Occupational Health (OH). Ecological and correlational study of the Relative Search Volume (RSV) obtained from Google Trends query, segmented in two searched periods concerning antiquity; date of query: 20 April 2021. The RSV trends for the analyzed three Topics were: Nutrition (R^2^ = 0.02), HD (R^2^ = 0.07) and OH (R^2^ = −0.72). There was a good positive correlation between Nutrition and OH (R = 0.56, *p* < 0.001) and a moderate one between HD and OH (R = 0.32, *p* < 0.001). According to seasons, differences were verified between RSV means in the Topics HD (*p* < 0.01) and OH (*p* < 0.001). Temporal dependence was demonstrated on Nutrition searches (Augmented Dickey–Fuller = −2.35, *p* > 0.05). There was only a significant relationship between the RSV Topic HD (*p* < 0.05) for the Developing and Least Developed countries. The data on the analyzed RSV demonstrated diminishing interest in the search information on HD and OH as well as a clearly positive trend change in recent years for Nutrition. A good positive correlation was observed between the RSV of nutrition and OH whereas the correlation between HD and OH was moderate. There were no milestones found that may report a punctual event leading to the improvement of information searches. Temporal dependence was corroborated in the RSV on Nutrition, but not in the other two Topics. Strangely, only an association was found on HD searches between the Developing and Least Developed Countries. The study of information search trends may provide useful information on the population’s interest in the disease data, as well as would gradually allow the analysis of differences in popularity, or interest even between different countries. Thus, this information might be used as a guide for public health approaches regarding nutrition and a healthy diet at work.

## 1. Introduction

Since the 1960s, the concept of health has shifted from a therapeutic to a preventive orientation. This change in philosophy, in conjunction with the rising costs of medical care, has spurred both individuals and their employers on searching for habits to improve welfare. In fact, there has been a high number of company organizations that have developed programs that stress the promotion of health. Evidently, most of these programs has included nutrition. Nutritional awareness, based on a healthy diet, is essential for achievement and health maintenance since adequate nutrition on its own does not assure good health. Certainly, this latter could not be achieved without the former [1].

Eating habits not only have to deal with the type of eating foods, but it also concerns the how, when, and where is eating. In short, they are habits and learned demeanors that can be taught and modified. Albeit it is important to bear in mind that many changes in diet are determined by the huge availability of advertising messages. On this matter, the World Health Organization document on “The workplace as a setting for interventions to improve diet and promote physical activity” indicated that the workplace provided a great advantage enabling them to achieve a large number of active people and, thus, to support the information and the training in nutrition and healthy diet of these workers [2].

Having said that, information plays a fundamental role in healthy food and a balanced diet. However, despite its importance, there is little information on employees´ attitudes, behaviors, and information preferences as well as vital details to targeted communication strategies. For this reason, it must ensure that commercial interests do not hamper occupational health policies and programs [3]. On the other hand, many people nowadays turn to the Internet for advice and resources related to their food. In fact, Doub et al., reported that up to 45% of the participants in their study announced having consulted websites to search for recipes concerning home cooking in the pursuit of a healthy diet [4].

Facing this situation, the idea that the population provides data on their tastes, searching services including the search on their disease athwart the search information behavior on Web has already been explored in recent years [5].

Google is an engine search that provides information to whatever person, which by means of the procured outcomes may readily access to the existed documents on the network. Google, which does not need presentation, was founded in 1997, and it is considered the most used search engine in the world with a market share that thoroughly overflows other search engines as Baidu o Yandex (the most used engines in China and Russia respectively) [6]. Although, Google Trends (GT) is not Google’s best-known implement, is a freely accessible tool that reports the volume of searches conducted by the users worldwide in order to expound on how frequently a term is searched and in what places. The search data on the Internet may provide helpful information on the demeanor population patterns [7,8].

In the health field, Eysenbach [9,10] coined the term “infodemiology” (information plus epidemiology) as an emerging set of public health information methods in order to analyze search behavior, communication and publication on the Internet. That is, observing and analyzing the behavior based on the Web to know the human demeanor with the purpose of foretelling, assessing and, even, preventing the health-related problems that constantly arise in the quotidian life [11].

Nowadays, in a marked scenario by the growing usage of social networks and interactivity resources, its inclusion in digital spaces for health communication becomes a relevant issue. Over the past decade, using Web-based data on health public issues in which infodemiology has shown to be useful for assessing various human demeanor features.

As Orduña-Malea [12] pointed out, it may be used in newcasting tasks, forecasting the present, that is, foretelling values that are occurring at the same time data is generating and it may be used as well in forecasting tasks (predicting future trends). Thus, this tool may facilitate to know many issues i.e., the most searched services, which are the new trends, or what needs are users demanding. GT is the indicated tool for gathering information and it has already been used in a wide range of topics hitherto, with studies in the field of diabetes [13], obesity [14], diet [15] and occupational health [16] among others.

As has been proved in recent years, these studies have shown the usefulness of knowing the habits, behaviors and interests of the general public. In this way, some prevention programs could be created regarding the topics studied.

Consequently, the aim of this paper was to study and relate the population interest athwart information search trends on Nutrition and Healthy Diet (HD) with the Occupational Health (OH).

## 2. Materials and Methods

### 2.1. Design

An ecological and correlational study of information search trends using Google.

### 2.2. Data Collection Search

The information search data was procured by means of direct search, online access, and Google Trends (GT): https://trends.google.es/, accessed on 3 December 2020. RSV data was downloaded directly from this platform. The CSV (Comma-separated Values) file of the results obtained was used for data collection, storage and subsequent analysis.

The scope was worldwide and in all categories. The study period was from 1 January 2004 to 30 June 2020. Searching and compiled data date were 20 April 2021.

To examine the evolution of the Relative Search Volume (RSV), the study time was divided into two periods: the first was from January 2004 (when GT provided the first data) until August 2012 and the second from September 2012 until April 2021.

### 2.3. Tool

GT is free and open access that provides standardized statistics of Google for different searches since 1 January 2004. It analyses the inquiries to delimit how many searches were carried out on a specific term in comparison with the total quantity of the conducted searches by users on Google for the same term and in the same period.

GT rules out terms with low volume search or duplicated searches performed by the same user in a short while.

### 2.4. Search Topic

Searches were conducted on the following topics: “Nutrition”, “Healthy Diet” and “Occupational Health”. The outcomes were taken from over the World and in all categories.

The results of the terms sharing the same concept in any language were obtained considering the use of the word as a "Topic" in this tool. (For example: if it is searched “Occupational Health”, the search includes results of the following topics “Job Security”, “Safety Committee” or “Health at Work”, among others).

### 2.5. Data Collection and Storage

The procured outcomes were downloaded in a standardized CSV format, which allowed them to be stored in a spreadsheet file. The quality control of this information was performed through double tables, amending the possible inconsistencies by consulting the original downloaded table.

### 2.6. Variables

Relative Search Volume (RSV): Result provided by Google Trends whose values are normalized on a scale from 0 (relative search volume less than 1% of the maximum volume) to 100 (relative search volume reaches its maximum). For instance, an RSV = 25 represents 25% of the highest observed search proportion during the period under study.

Trend: Temporal behavior and evolution of the searches carried out in a specific topic, long term.

Seasonality: Periodic and foreseeable variation in a time series with a period less than or equal to one year.

Unfolding level by country: An indicator created by the “United Nations Development Programme” that measure the breakthrough degree of each country dealing with variables such as life expectancy, education and per capita income. For its classification, the United Nations Statistics Division Website (UNdata Available online: http://data.un.org/, accessed on 16 November 2021) was consulted, which determines the three levels of development: Developed, Developing and Least Developed.

### 2.7. Procedure and Ethical Aspects

Anonymized data, which are available to the public, were downloaded from the Google Trends website (https://trends.google.es/, accessed on 3 December 2020). According to Spanish laws, Ethics Committee approval is not necessary if secondary data are being used.

### 2.8. Data Analysis

For quantitative data, its mean, Standard Deviation (SD), median, maximum and minimum were calculated. The Kolmogorov–Smirnov test (with Lillieforms correction) was used to verify the normality of the variables.

The Kruskal–Wallis test was employed to compare the medians among groups. The temporal evolution of search trends was analyzed by means of regression analysis calculating the coefficient of determination (R^2^). Pearson correlation coefficient (R) was employed to procure the relation amid quantitative variables.

The Augmented Dickey–Fuller (ADF) (tests the void hypothesis that an observable time series has a unit root against the alternative of stationary series) was used to verify seasonality.

The level of significance used in all the hypothesis tests was α ≤ 0.05, using asterisks to represent the strength of the association: * *p*-value < 0.05; ** *p*-value < 0.01; *** *p*-value < 0.001.

In most seasonality tests, the null hypothesis is the existence of a unit root that would rule out seasonality. This is the case of the Augmented Dickey–Fuller (ADF) test, one of the most widely used unit root tests today due to the robustness of its results. It uses an autoregressive model and optimizes an information criterion across multiple different lag values. The intuition behind a unit root test is that it determines how strongly a time series is defined by a trend. The null hypothesis of the test is that the time series can be represented by a unit root, that it is not stationary (has some time-dependent structure). The alternate hypothesis (rejecting the null hypothesis) is that the time series is stationary (and therefore does not depend on time).

The R software version 4.0.3 (Microsoft, Redmond, WA, USA) with the Rstudio work suite version 1.3.959 was used for data analysis.

## 3. Results

### 3.1. Relative Search Volume (RSV)

From the study of the procured data from GT it was verified that the Kolmogorov–Smirnov test discarded normality for all the subjects under study (“Nutrition”, “HD” and “OH”; *p* < 0.05), thus, we worked with nonparametric population comparison tests.

Central tendency statistics (mean and standard deviation, minimum and maximum) of the RSV values for each topic studied can be found in Table 1, along with their respective statistical results for the Kolmogorov–Smirnov normality test.

#### Correlation of RSV between the Topics

The association between the RSV data on Nutrition and OH demonstrated a good positive correlation (R = 0.56; *p* < 0,001), whereas between the HD and OH values were observed a moderate positive correlation (R = 0.32; *p* < 0.001).

### 3.2. Trends

**Figure 1:**
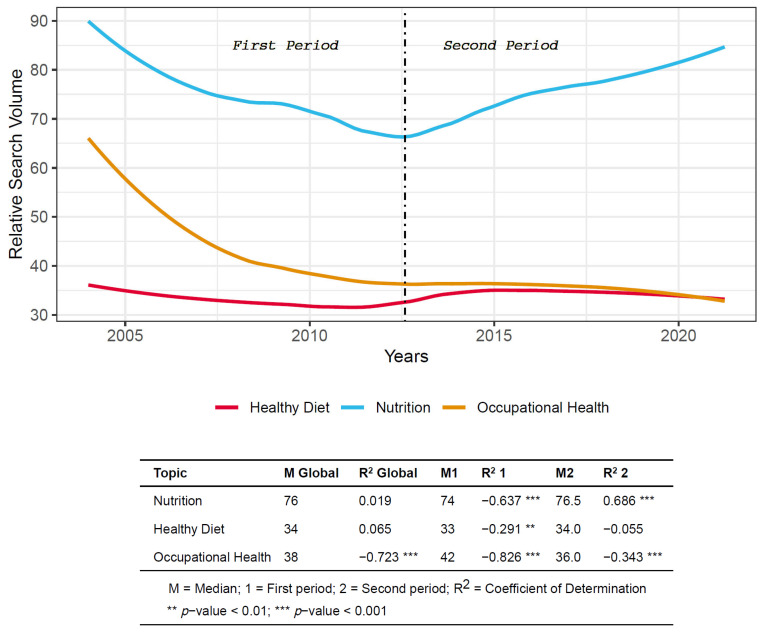
Search trends procured from Google Trends according to the 2 periods.

From the RSV data searches obtained from GT, the search trend was obtained eliminating the possible seasonality noise for the studied Topics (Figure 1). Along with this graphical representation, the median and the evolution (established by the coefficient of determination) are shown for the time series, as well as for each of the two analyzed periods.

**Figure 2:**
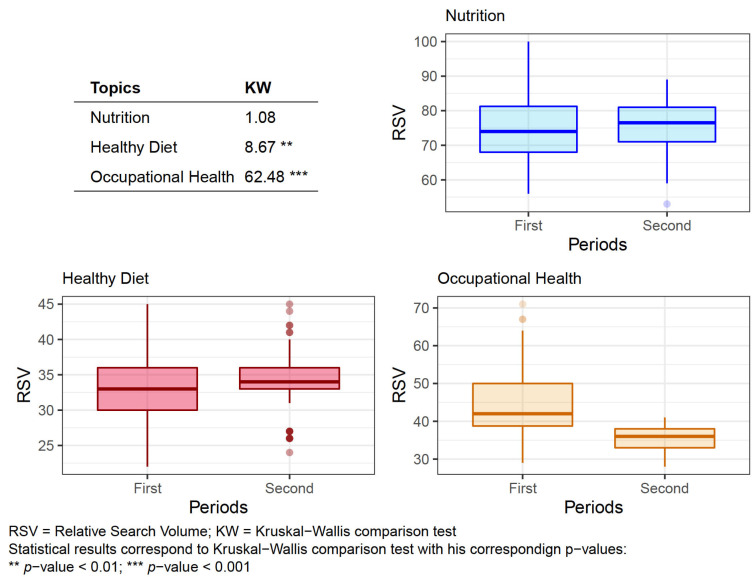
Box plots of the Relative Search Volume results of the analyzed Topics according to the different periods.

Having segregated the data according to the studied period, it was possible to calculate using the Kruskal–Wallis test, whether the RSV demonstrated the same distribution (Figure 2).

According to the two periods under study in the following topics: Healthy Diet (*p* < 0.01) and Occupational Health (*p* ≤ 0.001) significant differences were found in the RSV performance (of the searches), however, there was no significant differences in the searches on Nutrition.

The absence of landmarks (milestones) pointing out punctual events. Therefore, none of the performed searches highlighted a moment or circumstance that can give rise to the increase in queries related to the analyzed Topics.

### 3.3. Seasonality

**Figure 3:**
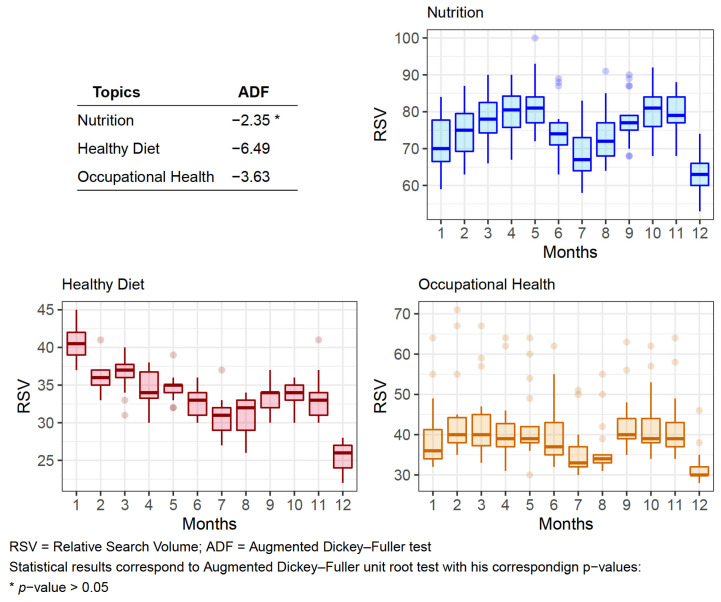
Box plots of the seasonality of each Topic grouped by month.

Throughout the years, the study seasonality in the searches was annually performed for the study period. Given the procured results using the specific and non-parametric test for seasonality, it has been possible to assert that only the Nutrition Topic showed temporal dependence which has not been presented in the other two Topics (HD or OH), see Figure 3.

The same test was performed segregating the countries by hemisphere, but the results obtained showed the same patterns as those shown in Figure 3. Indicating that there is not statistically significance in the seasonality separating by hemispheres. Likewise, a seasonality test was carried out, but with another time frequency (separated by the four seasons), and again, the results obtained were statistically the same as those shown in the global study.

### 3.4. Interest According to Country

**Figure 4:**
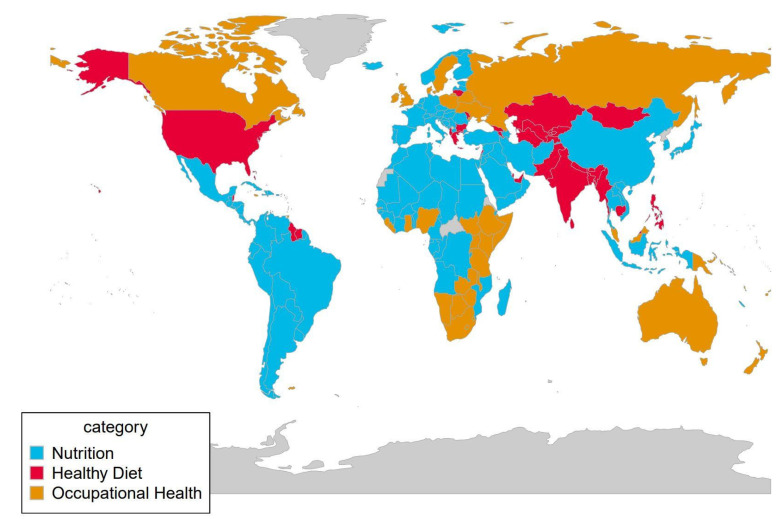
Most searched Topic by country.

The search interest in the topics studied by country can be seen in Figure 4, where the most searched topic prevails.

To determine the possible differences in population interest, procured from RSV, according to the level of development of the country, following the classification of the United Nations Development Program, a group comparison analysis was carried out for each topic.

According to this country classification, the central tendency statistics related to RSVs can be found in Table 2. The table shows the central tendency statistics (mean, median, minimum and maximum) for all the countries studied (Global section) and for each of the segregation groups (Developed, Developing and Least Developed countries).

**Figure 5:**
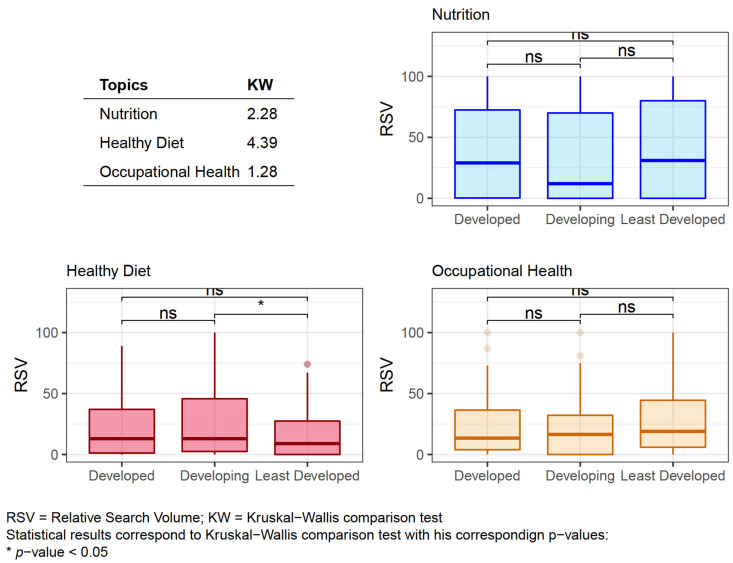
Box plots of the Relative Search Volume outcomes of the classified Topics according to the development level of the different countries.

No significant differences were found between the RSVs of each Topic except in the case of Healthy Diet where there was an association (*p* < 0.05) between Developing and Least Developed countries; see Figure 5.

## 4. Discussion

In this work, it has been verified that GT provides information on the population’s interest in health data and permits the analysis of differences in the popularity of certain Topics and even between different countries over time. In this infodemiological ecological study, the global popularity of searches on Nutrition, HD and OH related topics was ranked among Google users. The analysis disclosed some results that deserve a detailed discussion.

Nevertheless, it should take into consideration that this is an ecological data analysis, and the findings might not be representative at the individual level [17,18] (an ecological study is a type of epidemiological study based on the population as a unit of study in which information is lacking on the relationship in the individual between the exposure factor and the disease in the study population). For instance, RSV trends deal with the whole population, and it would be ill-considered to deduce that only individuals who have an interest in following a healthy diet are creating all the search volume. Undoubtedly, Internet searches have become the main source of health-related information, including Nutrition [19].

### 4.1. Relative Search Volume (RSV) and Trends

The RSVs on Nutrition present a nearly symmetrical graph concerning the two studied periods, which showed a clear positive trend in recent years. This situation is not observed in the other two Topics, HD and OH, which demonstrated a moderate negative trend, mainly in the second period. Possibly, as Modrego-Pardo et al. [20] commented the changing interest shown by the population in the different types of diets might influence the trend observed in HD.

Ayala-Aguirre et al. [21], pointed out that searching by using technical words was not common in Google Trends. Most of the population tends to search utilizing general topics or frequent words. Therefore, it would be easier to explain searches by using the following terms nutrition or occupational health than uncommon words i.e., healthy diet or another even less known type of diet.

The expected correlation between these Topics: Nutrition and HD with OH is due to the multiple research studies, which have concluded that dietary interventions in the workplace may have a positive impact on workers´ health [22]. In fact, nutritional intervention studies were conducted at work where significant changes were observed in worker´s eating behavior (increased consumption of fruit/vegetables, increased purchase of healthy options and reduce calories purchased) [23]. Thus, it is reasonable to believe that whatever intervention in favor of a healthy diet in the workplace will be reflected in employees´ interest in finding information on the subject.

It is worth indicating that the detection of unusual milestones of searches are usually related to the change in the normal course of disease [18] or might be a response to ad hoc information campaigns that often result in increased interest in seeking that information [24]. Nonetheless, it should be considered that this study is an ecological data analysis and the outcomes obtained might not coincide with the searches performed at the individual level, although it has been demonstrated that the concentration of news sways on public interest and perception, at a given time and for whatever reason [25]. Thus, the absence of milestones would imply that the population has not perceived any motivation to increase searches on the studied Topics.

It is relevant to point out the correlation among the topics studied. It was found that there is a good correlation between OH and Nutrition and a moderate correlation between HD and OH, suggesting that in both cases, public interest for one topic could be associated with the other.

### 4.2. Seasonality

In this study, seasonality has been analyzed as a fluctuating demand for a keyword (Topic) in the search engine throughout the year.

To verify the existence of epidemiological behavior, possible variations were studied in the searched information in which was corroborated that no Topic showed a temporal evolution (according to seasonality) in a sawtooth form [8]. These results discard that the analyzed searches have an “expected” behavior related to the time of year.

Although none of the topics presented an evolution of its RSV in a sawtooth form neither the existence of some highlighted milestone was corroborated, as has been stated in the previous section. Notwithstanding, a moderate seasonality was found for the Nutrition Topic. It is expected that the population’s diet including the employees varies at different seasons of the year which is manifested in the observed seasonality on Nutrition Topic.

The non-seasonality confirmed for the HD Topic was not anticipated because of the existing publications that have observed seasonality concerning a diet with a milestone in the spring months [19,26], this situation was not observed in the present study. Nevertheless, more studies on nutrition are required to elucidate the mechanisms that establish this seasonality [27].

It was not shown in the study that there were differences in the seasonality patterns between the two hemispheres. This may be because seasonality patterns could be more influenced by the Western holiday calendar, adopted in most countries regardless of their hemisphere, rather than by climatic or weather seasons.

On the other hand, the absence of any milestone regarding the searches on HD was also not expected since there is research that demonstrate the pandemic COVID-19 has brought about dietary changes during the confinement period with a tendency towards increased consumption of healthy food, decrease consumption of less nutritional food and the growing practice of cooking at home [28,29,30]. This issue has not been reflected in Google searches.

Even though, GT is considered a great promise as a timely, robust and suitable system surveillance. It is currently more practical for surveillance of high-prevalence diseases since, to be effective, it requires a large web search volume for obtaining clearly upward trends for the entire study time-period [31,32]. This situation was not presented in this work.

At any rate, the graphic model of the RSV is a visualization that allows recognizing the connection between the turns of dialogue in a colloquial conversation (whether verbal, written or digital) [33], as well as this graphic model, provides a cause-effect communication between the events concerning given Topic and the necessity for further information.

### 4.3. Interest According to Country

The RSV data obtained with GT facilitate to have an insight into the interest of the public, from different countries, in the topic under study. As it has been proved and according to previous work [21], search engine trends are a tool that may integrate into real-time the monitoring of the population’s health information needs.

According to the development level, it was unexpected the lack of the difference between the diverse groups of countries since it is well-known that the existence of the occupational epidemiological situation in the majority of developing countries is heavily influenced by nutritional problems [34]. In fact, it has been long known that the nutritional problems of developing countries are conditioned by poverty, the near-exclusive dependence on plants sources of nutrients and high infection rates [35]. Obviously, these circumstances influence occupational health. Moreover, occupational health is evidently a neglected problem in developing countries [36].

A study performed by the International Labour Office (ILO) [37], showed that a poor diet at work generates a loss above 20% of productivity for the whole countries around the world. This issue befalls either due to malnutrition that affects millions of people in developing countries or because of overweight and obesity that damage a similar number of people which mainly occurs in industrialized economies.

There is growing evidence that obesity and overweight might be partly related to adverse working conditions. Additionally, obesity may be a risk co-factor for the development of the following diseases: skeletal muscle injuries, occupational stress or cardiovascular diseases [38].

Nonetheless, the previous explanation has not been manifested in the RSV analysis, except in the found association on HD RSV between developing and less developed countries. Regardless, it would be more logical having observed this relationship between the developed and the rest countries.

However, when analyzing RSV´s, it should be taken into consideration that it is difficult to know the responsible relationship of outcomes by region regarding Google access (or any search engine) and these may be due to different reasons (appearance of news in the main media of a certain country, regional health campaigns focused on a certain disease and so on), and remember that GT only provides results of searches that have high traffic [8].

On the other hand, no country was found where the RSV of the Topics under study was not reported. This circumstance, if it was found, is mainly due to two motives: the digital divide or the filtering of social content. Regarding the digital divide, it is not only a technological problem, but also the differences that appear in having or not an Internet connection, or in the type of connection. Digital health services depend on sociodemographic and socioeconomic factors. The filtering of social content (censorship) befalls in countries for which Internet blocking is observed, censoring topics that contradict the accepted social rules of a country (pornography, gambling, alcohol, and drugs, LGTBI content and so on). These countries that are characterized by censoring online news and information are known as “enemies of the Internet” [39,40,41].

### 4.4. Limitations of This Study

According to Johnson and Mehta [17], given that Google Trends does not provide real usage data and more accurate time intervals, decreases the forecasting capability. Besides, greater transparency is lacking, since there is no information on the specific methods and models that Google uses to calculate the RSV, and as it has been suggested in several publications [8,18,42], the outcomes procured using this tool might be swayed by media interest, mainly advertising campaigns, which may not exactly correspond to the interest of the general population.

On the other hand, this research was based on the conducted searches through Google without considering other search engines. However, in 2020, this browser was in the first ranking with a global market share of more than 92% [43].

Another limitation of this study is the lack of articles that exist on the infodemiology field, that relate the studied topics of Nutrition and Occupational Health. In this way, the results obtained may represent a new source of data on population health and be used as a complement to standard disease surveillance systems [13,14,15], but currently they cannot replace conventional surveillance systems. More studies on the useful-ness and limitations of these methodologies are needed.

Finally, it must be acknowledged that this study has a limitation on the “connected world” and therefore, there will be a bias in terms of the results that can be extracted from the behavior patterns of the population.

## 5. Conclusions

The data on the analyzed RSV demonstrated diminishing interest in the search information on HD and OH as well as a clearly positive trend change in recent years for Nutrition. A good positive correlation was observed between the RSV of Nutrition and OH whereas the correlation between HD and OH was moderate indicating that there could be a mutual interest by the studied topics. There were no milestones found that may report a punctual event leading to the improvement of information searches. Temporal dependence was corroborated in the RSV on Nutrition, but not in the other two Topics. Strangely, only an association was found on HD searches between the Developing and Least Developed Countries.

The study of information search trends may provide useful information on the population’s interest in the disease data, as well as would gradually allow the analysis of differences in popularity, or interest even between different countries. Thus, this information might be used as a guide for public health approaches regarding nutrition and a healthy diet at work (interest in certain information, searches carried out on the Internet, together with other epidemiological indicators can be of great interest for certain health actions).

## Figures and Tables

**Table 1:** Descriptive statistics of the Topics under study and normality test.

Topic	Mean	Minimum	Maximum	Kolmogorov–Smirnov
Nutrition	75.32 ± 8.42	53	100	0.19 ***
Healthy Diet	33.67 ± 4.11	22	45	0.06 *
Occupational Health	40.12 ± 8.60	28	71	0.19 ***

Asterisks represent the strength of the association (* *p*−value < 0.05; *** *p*−value < 0.001).

**Table 2:** Central tendency statistics of the Relative Search Volume (RSV) according to country classification.

Country Classification	Topic	Mean	Median	Minimum	Maximum
Global	Nutrition	36.18	22	0	100
	Healthy Diet	23.34	13	0	100
	Occupational Health	23.06	16	0	100
Developed countries	Nutrition	37.64	29	0	100
	Healthy Diet	22.65	13	0	89
	Occupational Health	23.05	13.5	0	100
Developing countries	Nutrition	33.71	12	0	100
	Healthy Diet	26.32	13	0	100
	Occupational Health	21.22	16.5	0	100
Least Developed countries	Nutrition	40.85	31	0	100
	Healthy Diet	16.17	9	0	74
	Occupational Health	28.09	19	0	100

## Data Availability

The information search data was procured by means of direct search, online access, and Google Trends (GT): https://trends.google.es/, accessed on 3 December 2020. Relative Search Volume data was downloaded directly from this platform.
